# Effects of a Wireless Charging System Built for An Electric Kick Scooter on Human Biological Tissue

**DOI:** 10.3390/ijerph17082662

**Published:** 2020-04-13

**Authors:** Ibrahim Dergham, Yasser Alayli, Rodrigue Imad, Yskandar Hamam

**Affiliations:** 1 Laboratoire d’Ingénierie des Systèmes de Versailles(LISV), Paris SACLAY University, 78140 Vélizy, France; yasser.alayli@uvsq.fr; 2 Mechatronics Department, University of Balamand, Koura, Lebanon; rodrigue.imad@balamand.edu.lb; 3 Department of Electrical Engineering, Tshwane University of Technology (TUT), Pretoria 0001, South Africa; hamama@tut.ac.za; 4 ESIEE Paris, 93160 Noisy le Grand, France

**Keywords:** magnetic field, electrical field, wireless charging, human health, specific absorption rate, induced current, temperature increasing

## Abstract

In this paper, the authors present an evaluation of the electromagnetic fields generated by a static wireless charging system designed for an electric kick scooter on the human biological tissue. The guidelines on the exposure to the electromagnetic fields are previously specified. In this work, a model is designed under COMSOL multi-physics to study the effects of the magnetic field on two possible body parts of a person, which might be exposed to this field, namely the head and the hands is analysed. The magnetic flux density, the induced electrical field, the specific absorption rate, and the resulting increase of temperature of biological tissues are modelled and compared to the limits and guidelines prescribed in the regulation established to limit the exposure of people to electromagnetic fields. Furthermore, the used wireless charging system is modified to operate at higher frequencies to study its effect. The obtained results are below the guidelines and limits of exposure to the electromagnetic fields specified by the International Commission on Non-Ionizing Radiation Protection, European Commission, Institute of Electrical and Electronics Engineers and International Electrotechnical Commission.

## 1. Introduction

**Figure 1:**
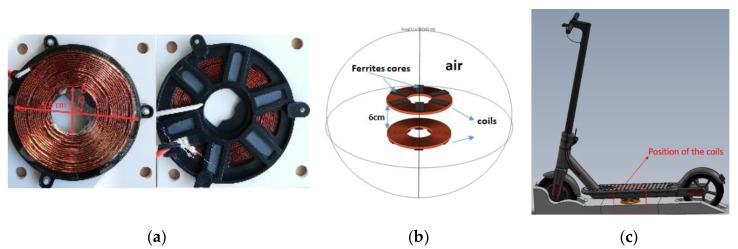
(**a**) Geometry of real coil; (**b**) COMSOL air domain and coils geometry; (**c**) position of the coils.

Electromagnetic fields (EMFs) exist in nature and have always been present among us. However, the increase in demand for electricity and developments in the field of wireless technologies (telecommunications, wireless networking, radio frequencies, wireless charging) have increased environmental and human exposure to sources of EMFs at different frequencies and field strengths. A new form of EMF sources has started to occur in the commercial and public market, which is in the form of high current induction chargers for electric vehicles, electric scooters, electric bicycles, and robots. Wireless charging is based on the phenomenon of induction demonstrated by Faraday in 1831, which states that when an electrical conductor is in a changing (alternating) magnetic field, an electromotive force is produced across the conductors. The concept of wireless charging technology relies on inductive coupling by means of electromagnetic fields that transfer energy through the air from the transmitter coil to the receiver coil. Figure 1a,b shows the real and the modelled coils, respectively.

As a result, the study of the effect of exposure to the electromagnetic fields on the health of humans is increasing with this technological evolution. Extensive research has been done on biological reactions due to overexposure of humans to electromagnetic fields, and the authors of references [1,2,3,4,5,6,7] present protective guidelines based on scientific evidence. Several national and international organizations (including the International Commission on Non-Ionizing Radiation Protection (ICNIRP) [1], the European Commission [2], and the Institute of Electrical and Electronics Engineers (IEEE) standard coordinating committee [4]) are reviewing the guidelines for the protection of people exposed to EMF at different frequencies. These guidelines are periodically revised and updated as advances in relevant scientific knowledge are made. There are two levels of protection, basic restrictions in terms of biologically effective quantities (induced current and Specific Absorption Rate (SAR)) and reference levels in terms of an external exposure metric (external magnetic and electric fields). However, if the reference level is exceeded, the basic restriction is not necessarily exceeded. Whenever the reference level is exceeded, it is necessary to verify compliance with basic restrictions [1,2,3,4,5]. Accordingly, these organisations have concluded by referring to peer-reviewed scientific journals that the exposure below the limits set out in the current guidelines do not seem to have any known detrimental effect on health. Scientific results on the effects of low-frequency magnetic fields are based on several epidemiological, animal, and in vitro studies. In reference [6], the authors highlighted many adverse health effects, ranging from neurological, neuroendocrine, neurodegenerative disorders to reproductive abnormalities to cardiovascular diseases. For high frequencies, the preponderance of radiofrequency field data suggests that exposure to low-intensity RF fields (such as those from mobile phones and their base stations) does not result in significant health effects. Some scientists have reported that the effects of mobile phone use can include minor changes in brain activity, reaction times, and disrupted sleep patterns [1,2,3,4,5,6,7].

A large number of researchers have reported low-frequency dosimetry (LF) [8,9,10,11,12,13,14]. Most of them approach uniform MF. Many numerical models have been used to study the effect of LF on human tissues such as the finite-difference time-domain (FDTD), scalar-potential finite difference (SPFD), finite-element method (FEM), boundary-element method (BEM), FEM/BEM, and method of moments (MoM). The adequacy of these methods is related to the complexity of the model and the heterogeneity of the electrical properties of the human body [15]. The numerical methods based on cube voxels such as FDTD, SPFD are the most used in dosimetry [15]. However, this method presents numerical errors due to stair-casing error. Many researchers have presented methods to reduce this error, such as smoothing [12], and the ICNIRP recommends using the 99th percentile value to calculate the induced field in the human body [1]. The other conformal methods using unstructured meshes such as FEM and BEM present an advantage of avoiding stair casing error but are less used in numerical dosimetry [15].

In this paper, the authors study the effects of a 160 W wireless charging system designed for the charging of an electric kick scooter on a persons’ head and hands by modelling using COMSOL software in order to determine the magnetic flux density, Specific Absorption Rate (SAR), and temperature elevation due to the magnetic field exposure.

This paper is organised as follows. Section 2 introduces the principles of the electromagnetic field, the heating mechanism, and a brief explanation about their effects on the human body. In Section 3, the COMSOL model is built to study the magnetic flux density, the induced electrical field, specific absorption rate (SAR), and the heat transfer in the head and hands. The results of the modelling are presented in Section 4. Finally, a conclusion of this work is given in Section 5.

## 2. Theoretical Background

The EMF phenomenon consists of electric and magnetic fields that vary or change with space and time. Their spatial variation depends on the electromagnetic properties of the material (the electrical permittivity and the magnetic permeability). Furthermore, an electric field (EF) is a vector field that exists by the presence of an electric charge and is expressed in volts meter (V·m^−1^), while a magnetic field (MF) is the physical result of the displacement of an electric charge, and it is expressed in two ways: magnetic flux density B in tesla (T) or magnetic field strength H in amperes/meter (A·m^−1^) with B = μH (μ being the magnetic permeability). The Maxwell equations describe the magnetic field and electrical field mentioned above. Furthermore, the electromagnetic fields can be divided into low frequencies (from 1 kHz to 100 kHz) and high frequencies (beyond 100 kHz) according to ICNIRP. Also, EMF is considered non-ionizing radiation as it is too weak to break the bonds that maintain the molecules in cells and, therefore, cannot produce ionization [1,6].

Table 1 describes the various quantities, their symbols, and their units used throughout this paper. The effect of exposure to EMF depends on its frequency and intensity or magnitude. At low frequency, the human body perturbs the electrical field but not the magnetic field, while human tissue behaves like air for the magnetic field, and therefore, the internal and external MF are approximate equal [1,16]. Therefore, a magnetic field generates an induced internal electrical field referring to Maxwell–Faradays’ Equation $\vec{Rot}$ $\vec{E}$ = $-\frac{\partial \vec{B}}{\partial t}$.

Accordingly, the current density induced in tissue by this internal electrical field is given by the differential form of Ohm’s law

(1) $$\vec{J}=\sigma \vec{E}$$

where J is current density, σ is the conductivity, and E is the induced electrical field. Its intensity depends on the magnetic flux density and the size of the loop through which the current flows. Thus, when these currents are large enough. They can cause unwanted stimulation of the nerves and muscles [1,2,6,16]. Referring to the ICNIRP guidelines for a very localised non-uniform MF at low frequencies, the physical quantity used to specify the basic restrictions on exposure to EMF is the internal electric field strength E [1], as it is the electric field that affects nerve cells and other electrically sensitive cells in the body.

### 2.1. Specific Absorption Rate (SAR)

The specific absorption rate or SAR is the quantity of energy dissipated per mass of human tissue and has a unit of watts per kilogram (W/kg). In addition, SAR is proportional to the square of the internal electric field, and it is related to it in material or biological tissue by Equation (2) according to references [1,2,4] and [17,18,19,20]:

(2) $$SAR=\sigma \frac{\left|E\right|2}{2\rho }\;=\sigma \frac{\left|E_{rms}\right|2}{\rho }$$

where E and $E_{rms}$ are the peak, and the root mean square value of the internal EF, σ is the conductivity of tissue, and ρ is its mass density.

When referring to the general guidelines of ICNIRP and the European directives cited in references [1,2] and [3], it is possible to differentiate between two values of SAR—whole-body SAR and spatial average SAR. The whole-body SAR is the energy absorbed by the whole body divided by its mass, while the spatial average SAR is the energy dissipated within a local region by averaging over specific volume or mass (generally 1 g or 10 g of tissues) [2,3].

### 2.2. Bio-Heat Transfer

Penne’s bio-heat equations predict the heat transfers in human tissue and include the effect of blood perfusion on tissue temperature [18,19].

The general form of Pennes’ Equation is written as

(3) $$\rho c\frac{dT}{dt}=\nabla (k\nabla T)+w_{b}c_{b}(T_{a}-T)+q_{m}$$

where ρ is the mass density, c is the specific heat capacity, *k* is the thermal conductivity, and $q_{m}$ is the metabolic heating. $c_{b}$, $w_{b}$, $T_{a}$ are the specific heat capacity, perfusion rate, and temperature of the blood, respectively. T is the temperature to be targeted. The value of these heat properties depends on each organ and based on the findings of [10,19,21,22].

### 2.3. Relation between SAR and Bio-Heat Transfer

When biological tissue is exposed to an external heat source $Q_{s}$, this source should be combined with the bio-heat equation [9,10]:

(4) $$\rho c\frac{dT}{dt}=\nabla (k\nabla T)+w_{b}c_{b}(T_{a}-T)+q_{m}+q_{s}$$

In our application, the external heat source is the MF, which introduces internal electrical field in the human tissue, thus, in turn, inducing current flow in the tissue as described in Equation (1).

The movement of current in biological tissue produces a heating effect represented by the differential form of the Joule heating relationship $\frac{dP}{dV}$ = $\frac{1}{\sigma }J^{2}$ [23].

By combining (1) and (2), we find

(5) $$Q_{s}=\rho SAR$$

Thus, the heat source due to external MF is equal to the mass density of the tissue multiplied by the SAR. Referring to guidelines, the effect of heat on exposure to an EMF becomes important for frequencies above 100 kHz.

## 3. COMSOL Model

In order to study the effect of our wireless charging system built for a kick scooter, a model is built under the finite element method (FEM) and multi-physics software package COMSOL Multi-Physics. Many models have studied the effect of a high power wireless charging system going from 3.2 kW to 20 kW for electric vehicles, where the systems are shielded to limit the leakage magnetic field. Besides, humans are not directly exposed to the source of the magnetic field due to the presence of the vehicle chassis [24,25,26]. However, in this paper, the authors have studied the effect of a 160 W wireless power transfer (WPT), where the human body can be directly exposed to the magnetic field between the two coils, and therefore, the magnetic field density at this position is the most important.

Here, the two coils of our 160 W induction charging system have been modelled in a finite element air sphere with two coils superimposed at a parallel distance of 6 cm. Both coils are constructed from copper with ferrite cores with 17 spires (inner radius = 2.25 cm, outer radius = 5.6 cm), as shown in Figure 1a,b. The transmitter coil is positioned under the chassis of the scooter, as shown in Figure 1c. The system is not shielded due to its adverse impacts on efficiency [25]. However, the system must be shielded when the MF evaluation exceeds the limit to mitigate it.

**Figure 2:**
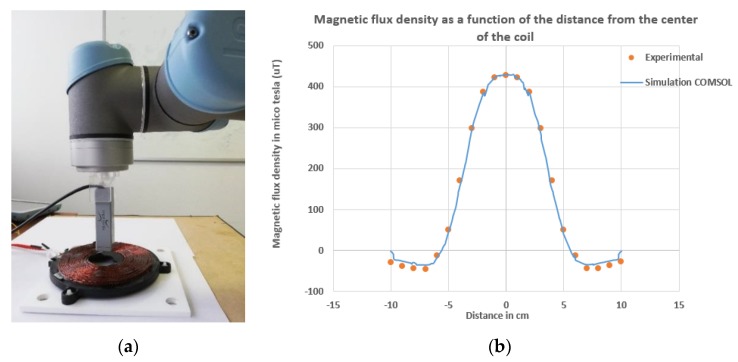
(**a**) measurement of magnetic flux density using magneto-resistive sensor; (**b**) experimental and simulation magnetic flux density as a function of distance.

Before simulating the complete model, a simple simulation is performed under COMSOL, in order to validate the geometry of the two coils used in our wireless charging system. Thus, a single-coil is modelled in a finite element air sphere, where a 2A current source powered the coil. The exact resolution of this system amounts to solving the magnetic vector potential and the Ampère–Maxwell equation [27,28,29]. Furthermore, the experimental measurement of the flux density of the magnetic field is done in order to validate the COMSOL physics study and the geometry of the two coils used in our simulation. To achieve this, one coil is introduced with a static current source of 2A, and the magnetic flux density is measured with magneto-resistive sensors, as shown in Figure 2a. The sensor is fixed using a universal robot at 2 cm above the centre of the coil. Thus, the flux density of the magnetic field is measured as a function of the x-axis.

Figure 2b shows the results of the simulation and experiment. It can be clearly seen that the simulated and the experimental graphs match with a small error for low densities of the magnetic field due to the resolution of the sensor. Thus we can validate the geometry of the coils and the physics used in our COMSOL model.

Furthermore, in order to study the effect of the magnetic field generated by our wireless charging system producing 160 W at a frequency of 30 kHz, two possible body parts of a person that might be exposed to the magnetic field, namely the head and the hands, were introduced. The human head is positioned in front of the two coils at a distance of 4 cm to mimic someone looking at the system from the side. The shape of the human head (with a skull partially removed) is taken from the standards of the IEEE, the International Electrotechnical Commission (IEC), and the European Committee for Electrotechnical Standardization (CENELEC) [30].

**Figure 3:**
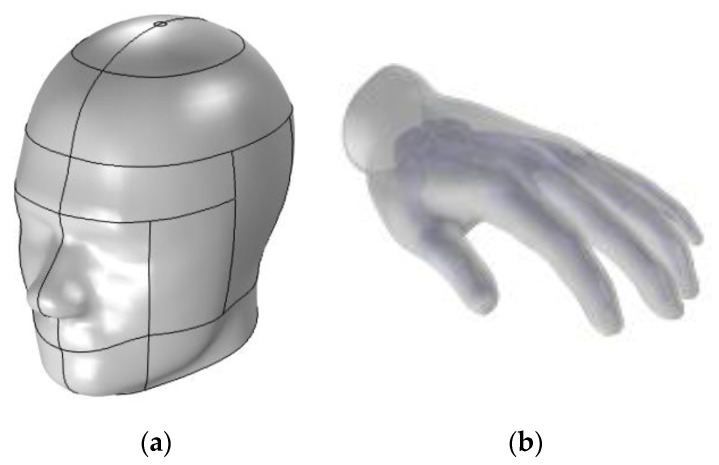
(**a**) Modeled head; (**b**) Modeled hand.

The model samples some organs material parameters (conductivity; permeability for skull, eyes, and brain) with a volumetric interpolation that assesses the variation of these parameters related to their positions inside the head based on references [31] and [32]. The carrying data file is created from a magnetic-resonance image (MRI), and its resolution is modified to correspond to that of our multi-physics model mesh, as shown in Figure 3a. According to the adult body size, a CAD model of a human hand is built base on the 3D morphology of referents [33]. The human hand of a 70 kg male is modelled with just two biologics tissues (skin and bone) as shown in Figure 3b with specific parameters shown in Table 2. The hand is positioned between the two coils in order to study the effect of a magnetic field if someone would try to put his hand between the two coils of the charging system. The skin is composed of multilayer tissues, which are the epidermis, dermis, and subcutaneous tissue. It is considered by the ICNIRP 2010 as one of the target tissues for the peripheral nervous system, and the ICES considers it as a topic to be solved. Previous literature has provided data on dielectric parameters of the skin [9,34]. In reference [35], the authors have provided new data on the conductivity of the tissue of the skin at frequencies. They found that the conductivity of the epidermis determines the conductivity of the skin. In our model, the conductivity of the skin is based on the findings in reference [35]. The thickness of the hand skin is taken from reference [36].

The reason we chose these two human limbs (head and hand) is to take two possible extreme cases of a human being in front of or near the induction charging system. The specific position of the head is chosen as it is the body part that contains the brain and is part of the very fragile central nervous system. It might be exposed to the MF when trying to look at or inspect the charging system from the side. The charging is made using a static wireless charging system. Thus, the charge is done without the presence of the driver. However, if someone stands on the scooter during charge, it is noticed that the density of the magnetic field near the body is less than the density between the two coils. Hence, this case is considered non-extreme.

## 4. Calculated Results and Discussion

In this section, the authors present the results of the simulated model. Then they compare them to guidelines as determined by the ICNIRP, the European Commission, and IEEE—ICES.

### 4.1. Magnetic Flux Density and Induced Current

**Figure 4:**
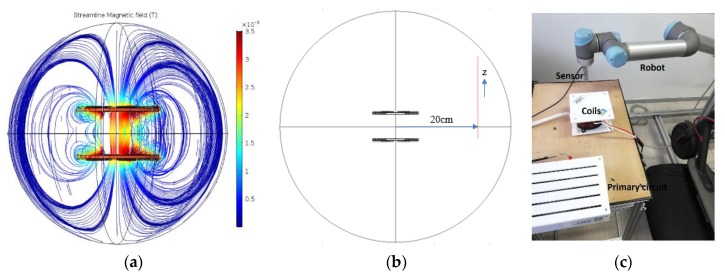
(**a**) Magnetic field streamline and its flux density in tesla (T) of 160 W wireless charging system at 30 kHz; (**b**,**c**) simulation and experimental models to measure the $B_{rms}$ of the magnetic flux density.

The shape of the magnetic field lines and their flux density ($B_{rms}$) for our wireless charging system are shown in Figure 4a. Referring to ICNIRP 2010 reference levels, the general public exposure to a magnetic field is limited to 27 µT for a range of frequencies between 3 kHz and 10 MHz [1].

Therefore, the root mean square of the flux density of the magnetic field $B_{rms}$ generated by a 160 W wireless charging system is measured at a position of 20 cm from the centre of the coils as a function of the z-axis (from z = 0 → 20 cm) as shown in Figure 4b. In fact, according to the ICNIRP guidelines, if the source of the magnetic field concern is localized at less than 20 cm from the body, the real evaluation of exposure to MF is to determine the induced electrical field in the body. When the distance exceeds 20 cm, the measure of the MF in the space occupied by the body is possible [1].

Figure 4b shows that the $B_{rms}$ curve does not exceed the limit recommended by the ICNIRP. We find the maximum $B_{rms}$ = 21.6 µT at z = 3.6 cm (opposite to the centre between the two coils). To validate the simulation, we measured experimentally the flux density of the magnetic field at the x, y, z-axis using the magnetoresistive sensor. Then, the $B_{rms}$ is calculated, as shown in Figure 4c.

**Figure 5:**
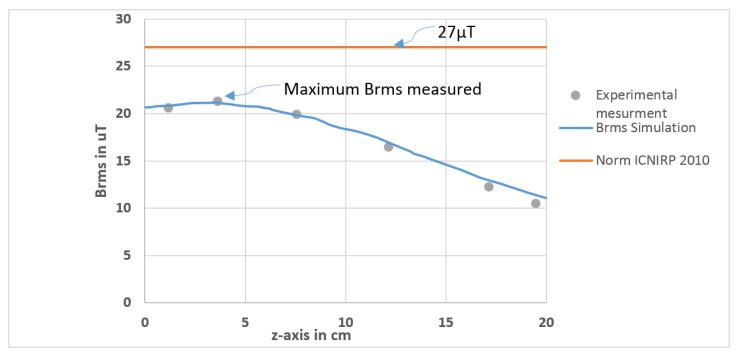
Simulation and experimental results of $B_{rms}$ comparing to the International Commission on Non-Ionizing Radiation Protection (ICNIRP) limit.

The result shown in Figure 5 validates that the experimental points coincide with the simulation curve and therefore, at this position, our system is safe, referring to ICNIRP reference levels.

In the next model, the authors aim to study the effects of a 160 W wireless charging system on human head and hand. Thus, the head is placed at 4 cm far from the system (MF very localized) and the hand between the coils, as presented in Section 3 and shown in the figures below.

Furthermore, in this model, the source of the magnetic field understudy is localized at less than 20 cm from the human body, so according to the ICNIRP guidelines, in this case, we determine the induced electrical field [1]. Thus, in the designed COMSOL model, the electrical field is calculated in a tetrahedral finite element with a volume of 7 × $10^{-8}\;m^{3}$ and 5.4 × $10^{-9\;}m^{3}$ for the head and the hand respectively. The calculated electrical field value is maximum local. Otherwise, for the ICNIRP, the internal electrical field is calculated as a vector average in a cube of 6 × $10^{-9}\;m^{3}$. The ICNIRP recommends considering the 99th percentile value as the maximum to filter the numerical stair casing error [1]. However, the 99th percentile is not accurate to localized exposure scenarios where the significant induced electric field is concentrated in a small volume [12]. The FEM method with unstructured mesh is chosen for its advantage against numerical methods based on cube voxels such as FDTD, SPFD to avoiding stair casing error [15]. Besides, unstructured mesh presents an advantage in modelling curved geometry such as the human body [37].

For a frequency of 30 kHz, the restriction of the induced electrical field is presented in Table 3.

**Figure 6:**
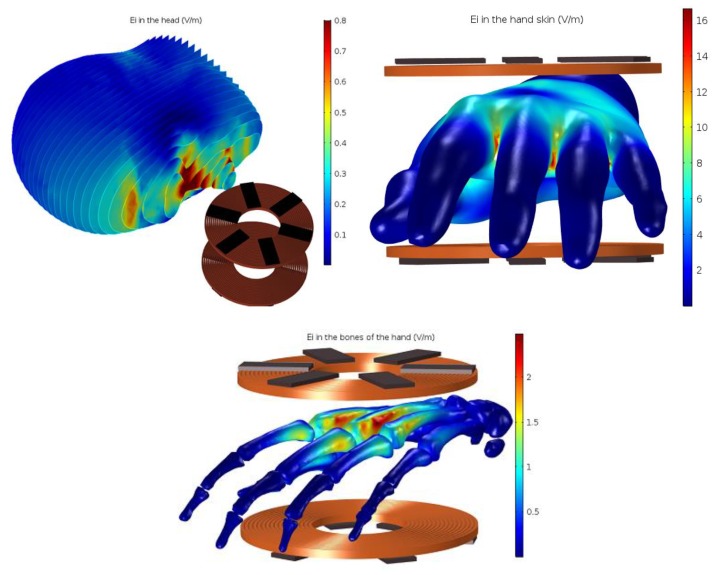
Induced electrical field in V/m of the head, skin and bones of the hand.

From Figure 6, it can be seen that the maximum internal electrical field strength (maximum $E_{rms}$) in the head is between 0.7 V·$m^{-1}$ and 0.8 V·$m^{-1}$ so at this value, the head is safe, referring to the restricted value of the induced electrical field represented in Table 3.

Furthermore, the maximum induced electrical field value in the skin of the hand is between 14 and 16.5 V·$m^{-1}$) and in the bones between 2 V·$m^{-1}$ and 3 V·$m^{-1}$ as shown in Figure 6. Thus, the result shows that the induced electrical field exceeds the restricted values for the skin, which can affect nerve cells referring to the ICNIRP and European commission. However, the hand is placed between the two coils; this is an extreme case, and it can be considered harmless to human health, and therefore, there is no need to shield the system.

### 4.2. SAR and Heat Transfer

#### 4.2.1. SAR and Heat Transfer at 100 kHz

In our simulation model, the Finite Element Method (FEM) is used to simulate the local SAR in a finite tetrahedral, although the guidelines have been specified using the Finite-Difference Time-Domain (FDTD) method to simulate the spatial average SAR by averaging the local SAR simulated in 1 g or 10 g of voxels cubes tissues [5].

The Federal Communications Commission (FCC) has ruled that FEM is a valid method to simulate the SAR as an alternative method to FDTD. This method is chosen for its advantages over FDTD for unstructured mesh and curved surfaces such as human organs [37]. Furthermore, the local SAR is generally greater than the 1 g or 10 g spatial averaging SAR, and when referring to basic restrictions, values of spatial averaging SAR are limited to 2 W·kg^−1^ in the head and 4 W·kg^−1^ in the limbs (general public from 100 kHz to 10 GHz). Our system operates at 30 kHz. However, the minimum frequency specified by the organizations to have a significant heating effect on human tissue is 100 kHz, so our SAR results are compared to this minimum frequency.

**Figure 7:**
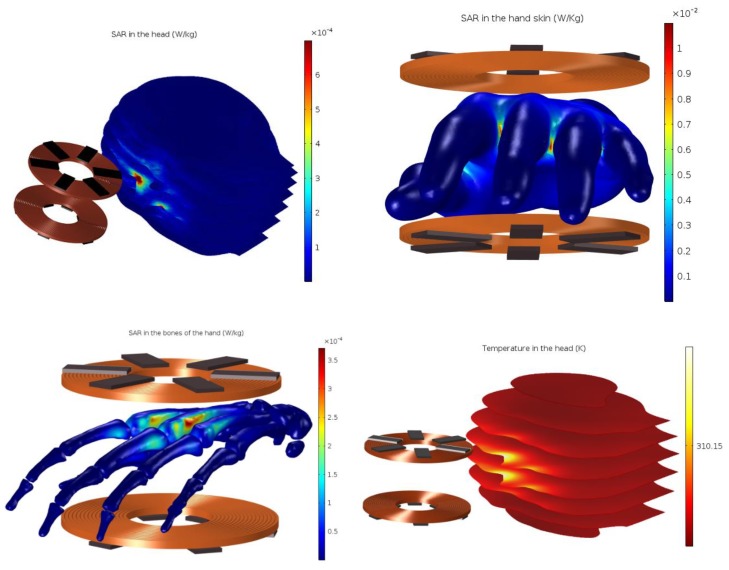
Specific absorption rate (SAR) in W/kg of the head, skin and bones of the hand; temperature increasing in the head.

Figure 7 shows that the local SAR in the head and the bones of the hand is in the order of $10^{-4},$ and in the skin is in the order of $10^{-2}$ which is significantly lower than the restrictions specified by the ICNIRP, the European Commission, and IEEE-ICES.

Moreover, in order to show the effect of the energy absorbed as a form of heat, we simulated the bio-heat transfer and SAR equations presented in Section 2 (the human being has been considered in a state of rest, and $q_{m}$ can therefore be neglected) [21]. The result of the temperature increase in the head is shown in Figure 7. The frequency-stationary study shows that the local increase of temperature in the head, skins, and bones at 30 kHz is equal to $10^{-6}$ K for the head and $10^{-5}$ K for the skin and bones, which is negligibly low and would be difficult to measure in practice, and will, therefore, be disregarded.

#### 4.2.2. Evolution of SAR and Temperature

In this part, the authors present SAR and temperature evolution with higher frequencies (100, 500, and 1000 kHz) for the head and the hand. Since the dielectric parameters of biological tissues change in terms of the frequency, the new dielectric parameters were modified for each frequency based on references [31,32].

**Figure 8:**
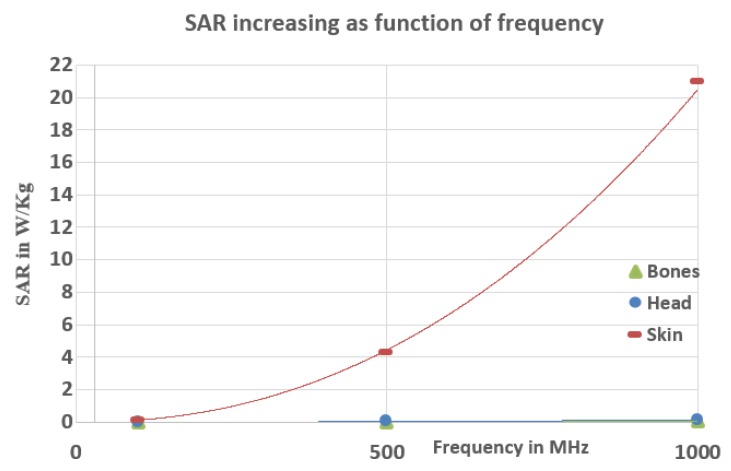
SAR increasing as function of frequency.

**Figure 9:**
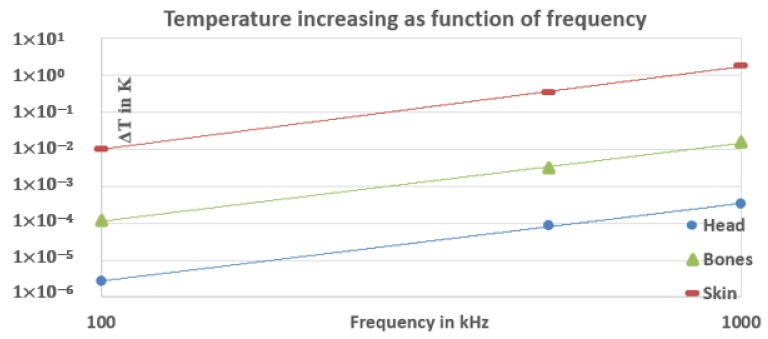
Temperature increasing as function of frequency (logarithmic scale).

Figure 8 and Figure 9 show the SAR and heat increase as a function of frequency. We can notice from Figure 8 that the SAR at 100 kHz in the head and the hand is order $10^{-1}$ which is less than the restrictions specified by the organizations. However, at 500 kHz and 1000 kHz, the SAR is still less than the restrictions for the head and the bones of the hand and much smaller than the skin SAR, which slightly exceeds the limit for 500 kHz (4.3 W/kg) and considerably exceeds for a frequency equal to 1 Mhz (21 W/kg). Therefore, it can be concluded that our wireless charging system at these frequencies (500 kHz and 1 MHz) starts to have a significant heat effect where the maximum local increase of temperature is equal to 1.81 °K at 1 MHz in the skin of the hand, as shown in Figure 9.

## 5. Conclusions

The authors have presented in this paper the magnetic effect of a 160 W static wireless charging system of an electric kick scooter on human tissues. Four parameters (magnetic flux density, induced electrical field, SAR, and heat) have been simulated and compared to the guidelines and limits specified by the ICNIRP, the European Commission, and IEEE-ICES. From the simulated results, our study has shown that the used wireless charging system may be considered harmless at 30 kHz, and the results of simulation of the four parameters are lower than the limits specified by the organizations, except for induced EF in the skin. By increasing the frequency, we got a bigger SAR and heat effects, which exceeded the skin the limits specified by the ICNIRP, the European Commission, and IEEE-ICES. Furthermore, high-power wireless transfer systems (3.6 kW, 7.2 kW, 11 kW) for electric cars are starting to be commercialized. In a few years, wireless systems are predicted to be everywhere—at home, work, in public places—and therefore, we will be exposed to more and more electromagnetic fields. Our future work will be further developed to determine the effects of this increase in the wireless system and electromagnetic fields on human health.

## Figures and Tables

**Table 1:** Units for magnetic and heat properties.

Symbol	Quantity	Unit
B	Magnetic flux density	T
J	Current density	A/m^2^
E	Electrical field strength	V/m
µ	permeability	H/m
σ	conductivity	S/m
ρ	Mass density	kg/m^3^
c	Heat capacity	J/(kg·K)
k	Thermal conductivity	W/(m·K)
$\mu_{r}$	Relative permeability	
$\varepsilon_{r}$	Relative Permittivity	
$T_{a}$	Arterial temperature	K
$c_{b}$	heat capacity of blood	J/(kg·K)
$w_{b}$	blood perfusion rate	kg/(s·m^3^)
$q_{m}$	Metabolic heat	W/m^3^

**Table 2:** Dielectric properties of the skin and bones of the hand at 30 kHz and 100 kHz.

Tissues	$\mu_{r}$	$\varepsilon_{r}$	σ	*ρ*
Skin (30 kHz)	1	1.13 × $10^{3}$	0.095	1090
Bones of arms (30 kHz)	1	308	2.06 × $10^{-2}$	1920
Skin (100 kHz)	1	1.12 × $10^{3}$	0.1	1090
Bones of arms (100 kHz)	1	228	2.08 × $10^{-2}$	1920

**Table 3:** Basic restrictions for electrical induced field E.

Frequency 30 kHz	$E_{rms}$
All tissues of head and body (ICNIRP 2010)	4.05 V/m
Entire body(European commission)	8.06 V/m

## References

[1] International Commission on Non-Ionizing Radiation Protection (2010). Guidelines for limiting exposure to time-varying electric, magnetic, and electromagnetic fields (1 Hz to 100 kHz). Health Phys..

[2] (2015). Non-Binding Guide to Good Practice for Implementing Directive 2013/35/EU Electromagnetic Fields.

[3] International Commission on Non-Ionizing Radiation Protection (2009). Guidelines for limiting exposure to time-varying electric, magnetic and electromagnetic field (100 kHz TO 300 GHz). Health Phys..

[4] IEEE (2010). Recommended Practice for Measurements and Computations of Electric, Magnetic and Electromagnetic Fields with Respect to Human Exposure to Such Fields, 0 Hz to 100 kHz, IEEE Std C95.3.1-2010.

[5] IEEE (2006). IEEE Standard for Safety Levels with Respect to Human Exposure to Radio Frequency Electromagnetic Fields, 3 kHz to 300 GHz, IEEE Std C95.1™-2005.

[6] World Health Organization (2002). Department of protection of the human environment. Establishing a dialogue on risks from electromagnetic fields. Radiation and Environmental Health.

[7] World Health Organization (2013). Non-ionizing radiation, part 2: Radiofrequency electromagnetic fields. IARC Monographs.

[8] Chan K.H., Hattori J., Laakso I., Hirata A., Taki M. (2013). Computational dosimetry for grounded and ungrounded human models due to contact current. Phys. Med. Biol..

[9] De Santis V., Chen X.L., Laakso I., Hirata A. (2015). An equivalent skin conductivity model for low-frequency magnetic field dosimetry. Biomed. Phys. Eng. Express.

[10] Hirata A., Yamazaki K., Hamada S., Kamimura Y., Tarao H., Wake K., Suzuki Y., Hayashi N., Fujiwara O. (2010). Intercomparison of induced fields in Japanese male model for ELF magnetic field exposures: Effect of different computational methods and codes. Radiat. Prot. Dosim..

[11] Dimbylow P. (2005). Development of the female voxel phantom, NAOMI, and its application to calculations of induced current densities and electric fields from applied low frequency magnetic and electric fields. Phys. Med. Biol..

[12] Laakso I., Hirata A. (2012). Reducing the staircasing error in computational dosimetry of low-frequency electromagnetic fields. Phys. Med. Biol..

[13] Neufeld E., Iacono M.I., Akinnagbe E., Wolf J., Oikonomidis I.V., Sharma D., Wilm B., Wyss M., Jakab A., Cohen E. Computational platform combining detailed and precise functionalized anatomical phantoms with EM-Neuron interaction modeling. Proceedings of the 2014 XXXIth URSI General Assembly and Scientific Symposium (URSI GASS).

[14] Bakker J.F., Paulides M.M., Neufeld E., Christ A., Chen X.L., Kuster N., van Rhoon G.C. (2012). Children and adults exposed to low-frequency magneticfields at the ICNIRP reference levels: Theoretical assessment of the induced electric fields. Phys. Med. Biol..

[15] Poljak D., Cvetkovi´c M., Bottauscio O., Hirata A., Laakso I., Neufeld E., Reboux S., Warren C., Giannopolous A., Costen F. (2017). On the use of conformal models and methods in dosimetry for nonuniform field exposure. IEEE Trans. Electromagn. Compat..

[16] Lin J.C. (2011). Electromagnetic fields in biological systems. IARC Monographs.

[17] Poljak D. (2018). Numerical Methods and Advanced Simulation in Biomechanics and Biological Process.

[18] Lakhssassi A., Kengne E., Semmaoui H. (2010). Modifed pennes’ equation modelling bio-heat transfer inliving tissues: Analytical and numerical analysis. Nat. Sci..

[19] Ji X., Zheng J., Yang R., Kainz W., Chen J. (2019). Evaluations of the MRI RF-induced heating for helical stents under a 1.5T MRI system. IEEE Trans. Electromagn. Compat..

[20] Wen F., Huang X.L. (2017). Human exposure to electromagnetic fields from parallel wireless power transfer systems. Int. J. Environ. Res. Public Health.

[21] Ramsey J.D., Bernard T.E., Dukes-Dobos F.N. (2000). Evaluation and Control of Hot Working Environments: Part I—Guidelines for the Practitioner. Int. J. Ind. Ergon..

[22] Ahmadikia H., Moradi A., Fazlali R., Parsa A.B. (2012). Analytical solution of non-fourier and fourier bioheat transfer analysis during laser irradiation of skin tissue. J. Mech. Sci. Technol..

[23] Marinova I., Mateev V. (2010). Electromagnetic field modeling in human tissue. Int. J. Biomed. Biol. Eng..

[24] Ding P., Bernard L., Pichon L., Razek A. (2014). Evaluation of electromagnetic fields in human body exposed to wireless inductive charging system. IEEE Trans. Magn..

[25] Mohammad M., Wodajo E.T., Choi S., Elbuluk M. (2019). Modeling and design of passive shield to limit EMF emission and to minimize shield loss in unipolar wireless charging system for EV. IEEE Trans. Power Electron..

[26] Wang Q., Li W., Kang J., Wang Y. (2019). Electromagnetic safety evaluation and protection methods for a wireless charging system in an electric vehicle. IEEE Trans. Electromagn. Compat..

[27] Touazi A., Dergham I., Alayli Y., Chassagne L., Linares J. (2018). COMSOL simulation of an attenuated magnetic field through a metallic plate. J. Phys. Conf. Ser..

[28] Anele A.O., Hamam Y., Alayli Y., Djouani K. Computation of the mutual inductance between circular filaments with coil misalignment. Proceedings of the IEEE Africon.

[29] Anele A., Hamam Y., Chassagne L., Linares J., Alayli Y., Djouani K. (2015). Computation and experimental measurements of the magnetic fields between filamentary circular coils. Int. J. Phys. Chem..

[30] Lee A., Hong S., Choi H. (2019). Is the SAM phantom conservative for SAR evaluation of all phone designs?. ETRI J..

[31] Gabriel C., Gabriel S., Corthout E. (1996). The dielectric properties of biological tissues: II. Measurements in the frequency range 10Hz to 20 GHz. Phys. Med. Biol..

[32] Hasgall P.A., Di Gennaro F., Baumgartner C., Neufeld E., Lloyd B., Gosselin M.C., Payne D., Klingenböck A., Kuster N. (2018). IT’IS Database for Thermal and Electromagnetic Parameters of Biological Tissues; Version 4.0.

[33] Lifesciencedb, BodyParts3D, Anatomography. https://lifesciencedb.jp/bp3d/.

[34] Schmid G., Cecil S., Uberbacher R. (2013). The role of skin conductivity in a low frequency exposure assessment for peripheral nerve tissue according to the ICNIRP 2010 guidelines. Phys. Med. Biol..

[35] Wake K., Sasaki K., Watanabe S. (2016). Conductivities of epidermis, dermis, and subcutaneous tissue at intermediate frequencies. Phys. Med. Biol..

[36] Oltulu P., Ince B., Kokbudak N., Findik S., Kilinc F. (2018). Measurement of epidermis, dermis, and total skin thicknesses from six different body regions with a new ethical histometric technique. Turk. J. Plast. Surg..

[37] Federal Communications Commission (2011). FCC-11-125.

